# Feasibility, usability, and acceptability of the asynchronous trauma-focused CARE training program for healthcare providers: a cross-sectional study

**DOI:** 10.1186/s12909-026-08794-8

**Published:** 2026-02-18

**Authors:** Dana C. Ross, Nancy McCallum, Aysha Butt, Annie K. Truuvert, Eileen Wang, David Rojas, Sophie Soklaridis, Simone N. Vigod

**Affiliations:** 1 Women’s College Hospital and Research Institute, Toronto, ON Canada; 2 Faculty of Medicine, University of Toronto, 1-2,6-8 Temerty, Toronto, ON Canada; 3 Health Sciences Program, McMaster University, Hamilton, ON Canada; 4 The Wilson Centre, University of Toronto, Toronto, ON Canada; 5 Centre for Addictions and Mental Health, Toronto, ON Canada

**Keywords:** E-health, Virtual, Psychoeducation, adverse childhood experiences, Complex trauma, Program evaluation

## Abstract

**Background:**

The long-term effects of adverse childhood experiences, including various forms of abuse, neglect, and trauma, are well-documented but often inadequately addressed in healthcare settings. Healthcare providers frequently lack the training necessary to provide trauma-focused, evidence-based interventions. To address this gap, we developed the ‘Community Access to Resourced & Resilient PsychoEducation’ (CARE) training program, a scalable, asynchronous, virtual learning platform designed to equip healthcare providers with the skills and knowledge to facilitate an 8-week trauma-focused psychoeducational group intervention for adults with a history of childhood interpersonal trauma.

**Methods:**

In this cross-sectional study, we evaluated the feasibility, usability, and acceptability of the CARE training program among 62 healthcare providers from six organizations across Ontario. Pre- and post-training questionnaires were used to assess these domains using a combination of Likert-scale items and open-ended questions.

**Results:**

Sixty-two healthcare providers from six Ontario organizations enrolled in the CARE training program; 42% had worked in the healthcare field for 11 or more years, and nearly all (96.8%) had prior experience with e-learning. 73% completed at least half of the modules and 65% completed ≥ 90%. Most participants reported completing all eight modules within 5–10 h. Among the 34 providers who completed the post-training survey, 97% agreed that the time required was well matched to the knowledge gained, and 97% reported that the multimedia format enhanced their learning experience. Almost all respondents described the modules as clear, practical, and easy to navigate, and most said they would recommend the training to colleagues. The most commonly reported barrier was finding time to complete the modules, while content on emotion regulation and trauma-related interpersonal patterns was highlighted as particularly useful.

**Conclusions:**

Our findings underscore the feasibility, usability, and acceptability of a virtual, asynchronous trauma-focused psychoeducational training program for healthcare providers. The CARE program’s multimedia, self-paced format was well received and offers a scalable approach to trauma-focused training across diverse healthcare settings. Future phases of this study will evaluate training-related knowledge and confidence and examine effectiveness and implementation outcomes.

**Supplementary Information:**

The online version contains supplementary material available at 10.1186/s12909-026-08794-8.

## Introduction

 The lasting mental and physical effects of adverse childhood experiences (ACEs), including various forms of abuse, abandonment, and neglect, have been extensively studied and recognized [1, 2]. However, the impact of trauma is often overlooked and inadequately addressed in healthcare settings [3, 4]. Compounding this, healthcare providers (HCPs) frequently lack the training and confidence to provide evidence-based therapy interventions for trauma [5, 6]. Limited access to affordable and broadly accessible trauma training programs contributes to this gap, as many existing options are intensive, modality-focused, and primarily aimed at mental health clinicians, making them difficult for many HCPs to access [7]. A possible solution is a scalable, virtual training program for HCPs, focusing on facilitating a psychoeducational group therapy intervention for individuals with a history of ACEs. Such a program could significantly increase the capacity of the healthcare system to provide trauma care for patients [8, 9].

The benefits of virtual training programs include increased cost-effectiveness, enhanced accessibility, and rapid knowledge transfer [10, 11]. Research shows that online learning is at least as effective as in-person methods for augmenting HCPs knowledge, attitudes, and confidence [12–14]. The flexibility inherent in asynchronous (self-paced) training formats presents a promising avenue for vastly expanding educational opportunities for HCPs [15, 16]. However, research is needed to evaluate how to optimize the design and implementation of such training programs across diverse healthcare education contexts [17, 18].

Evidence-based trauma-focused psychotherapies that are commonly used to treat trauma-related conditions often require extensive training to master, which poses challenges related to cost and time constraints for HCPs [19, 20]. In addition, although some HCPs can provide psychotherapy within their scope of practice, many do not have access to extensive trauma-specific training or the institutional resources needed to deliver intensive trauma-focused therapies. HCPs across diverse treatment settings may benefit from more broadly applicable treatment interventions that are both feasible for HCPs to deliver and efficacious for diverse populations impacted by trauma [21–24]. A stepped-care approach to mental health treatment, which has gained traction in the literature, offers a promising solution. Stepped care approaches typically match intervention intensity to client needs, offering lower-intensity options as an initial step before progressing to more specialized treatments. This approach requires less intensive training for HCPs and emphasizes lower-intensity interventions initially, focusing on psychoeducation and skills-building, which have demonstrated promising outcomes [25–27].

Importantly, psychoeducation as a treatment intervention for patients has demonstrated effectiveness in treating depression, anxiety, and complex mental health conditions like schizophrenia and bipolar disorder [28–30]. Psychoeducation aims to enhance patients’ knowledge and understanding of their struggles through education, insight-building, and skill development [31, 32]. It offers benefits such as improved treatment adherence, enhanced mood and self-regulation, reduced hospitalization rates, and symptom improvement [33, 34]. In addition, psychoeducational interventions are often offered in group settings, which can potentially be less resource-intensive and more cost-effective [34–37].

Based on the results of an earlier needs assessment study by our team, we developed the asynchronous, virtual ‘Community Access to Resourced & Resilient PsychoEducation’ (CARE) training program. The goal of the CARE training program is to equip HCPs with the skills and knowledge necessary to deliver an 8-week group-based psychoeducational treatment intervention for adult survivors of childhood trauma. The aim of current study, however, was not to evaluate the delivery of the group, but to assess the feasibility, usability, and acceptability of the CARE training program itself. By leveraging a virtual platform and asynchronous learning strategies, the CARE training program could overcome geographical distance and scheduling conflicts, increasing HCPs’ access to trauma training.

## Methods

### The intervention

**Fig. 1:**
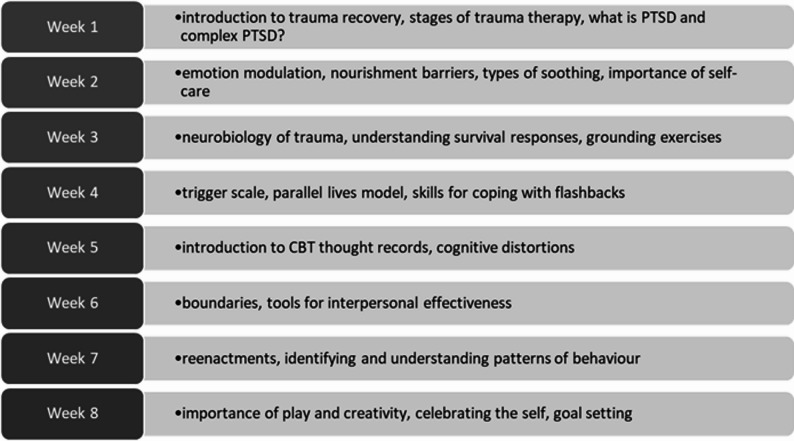
CARE Training Module content outline

The virtual, asynchronous CARE training program features eight modules based on content from an 8-week psychoeducational, trauma-focused Resourced and Resilient (R&R) group offered to adults enrolled in the Trauma Therapy Program (TTP) at Women’s College Hospital (WCH) in Toronto, Ontario, Canada. The TTP is in an academic ambulatory hospital department of the psychiatry program and offers primarily group psychotherapy to adults experiencing symptoms related to adverse childhood experiences. The R&R group was initially developed in 2004 by a multidisciplinary team of therapists and is a manualized group psychotherapy intervention that combines psychoeducation on the impact of childhood trauma, along with skills and strategies for healing. R&R is currently offered either in-person or synchronously via Zoom. It typically has 10–12 group participants and is co-facilitated by two staff members. The R&R group format was recently adapted in TTP to be a hybrid treatment intervention including self-paced online modules along with 1-hour weekly group sessions facilitated by trauma therapists [38]. This adapted version was evaluated in a pilot study showing a significant pre-post decrease in PTSD symptoms following completion of the intervention [39] and a randomized wait-list controlled trial has recently been completed [40]. The R&R group presents material focused on conceptualizing symptoms and struggles as understandable and often adaptive impacts of childhood trauma, while supporting the development of safer and more effective coping capacities. The content draws on evidence-based approaches shown to improve mood, anxiety, PSTD symptoms, self-regulation, and distress tolerance, including psychoeducational interventions [41–43], cognitive and dialectical behavioural therapy tools [44–46], mindfulness [47] and sensorimotor psychotherapy-informed strategies [48]. Additional detail is outlined in Fig. 1.

### Setting and participants

This cross-sectional study, using a pre-training questionnaire and a separate post-training questionnaire (supplementary file 1), was conducted at Women’s College Hospital between January 2020 and March 2021. HCPs were eligible for study participation, including social workers, physicians, nurses, registered psychotherapists, community support workers and mental health counsellors working at the six participating community healthcare organizations in Ontario, Canada. These six organizations had independently emailed the TTP program and expressed an interest in receiving trauma-related training. A research staff member then emailed organization contacts about the study. Interested individual HCPs from the participating sites responded via phone or email to learn more about the study and register for the asynchronous online CARE training program. Informed consent was obtained from participants. Once enrolled, a pop-up explaining the study, risks and benefits were presented to participants who were asked to select “I agree” to continue their participation in the study. Participant eligibility included having access to an internet-enabled device and the ability to speak and understand English. CARE training program registration was free, and participants were given approximately three months to complete the training modules. The team tracked participant progress and module completion.

### Data collection procedures

Data were collected using electronic questionnaires embedded within the Thinkific learning platform, which hosted the CARE training modules. Participants completed the pre-training questionnaire immediately after registering for the CARE program and before beginning module one. The pre-training questionnaire assessed prior training and experience with virtual courses. The post-training questionnaire was presented at the end of the final module, and evaluated feasibility, usability, and acceptability of the CARE modules.

Survey items were developed by the study team and reflected domains commonly evaluated in implementation science studies of digital healthcare interventions, including feasibility, usability, and acceptability studies [49–51], as well as content areas relevant to the CARE curriculum. Both Likert-scale items and open-ended questions were included. No personal identifiers were collected. Responses informed future refinements to the training content and overall platform design.

### Data analysis

Consistent with prior implementation science literature, feasibility was defined as the practicality of delivering the CARE training program as intended, including participant engagement, module completion, and time required to complete the training [52]. Usability captured the ease of navigation, clarity, and functionality of the online training program [53]. Acceptability referred to participants’ perceptions of the training content, including its relevance, value, and overall satisfaction [54].

To assess feasibility, we calculated the proportion of participants who completed the pre-training questionnaires and the proportion of participants who completed (a) all training modules and (b) at least 50% of the modules. Feasibility was also assessed by calculating the length of time it took to complete the modules. Usability and acceptability outcomes were calculated using Likert-type scale responses and through qualitative feedback from open-ended questions. All analysis were descriptive and conducted using Microsoft Excel.

## Results

### Participant characteristics

Sixty-two participants were enrolled in the study, completed the pre-module questionnaires, and answered questions about their experience with e-learning and trauma-focused training prior to module completion (see Table 1). The timeline for training program registration varied between sites, beginning in March 2020, with the last registration in December 2020. Additional demographic data was not collected but will be collected in an upcoming study phase examining knowledge outcomes following CARE training program completion.

**Table 1:** Participant characteristics

Characteristic	Number of Participants *n* = 62 (%)
Time working in the healthcare field	
Less than 1 year	13 (21.0%)
1–5 years	14 (22.6)
6–10 years	9 (14.5)
11–15 years	9 (14.5)
16 or more years	17 (27.4)
Time working in current position	
1–5 years	44 (71.0)
6–10 years	10 (16.1)
10–20 years	8 (12.9)
Done e-learning modules in the past	
Yes	60 (96.8)
No	2 (3.2)
Previous training on delivering trauma-focused health care	
Yes	49 (79.0)
No	13 (21.0)

### Feasibility

Regarding training program completion, 73% of participants completed at least 50% of the modules, and 65% completed the full training (i.e., at least 90% of the modules) (Table 2).

**Table 2:** Average time spent to complete all 8 modules *n* = 34(%)

2–4 h	6 (17.6%)
5–7 h	12 (35.3%)
8–10 h	10 (29.4%)
More than 10 h	6 (17.6%)

Out of the 62 participants, 81.3% of respondents took less than one hour to complete each module, and 18.8% took more than 60 min. 64.7% of participants required 5–10 h to complete all eight modules.

### Post-module evaluation

Of the 40/62 (65%) participants who completed at least 90% of the modules, 34 (85%) completed the post-training modules questionnaire (Table 3). Of these, 97% agreed that the time invested in completing the modules was well-matched to the knowledge gained and would recommend the modules to their colleagues. All respondents thought the content quality was consistent and clear, and the modules were easy to navigate, met their expectations, and had no significant technical difficulties. In particular, 97% felt the multimedia format improved their learning experience. No participants reported accessibility issues.

**Table 3:** Post-Training survey: usability & acceptability ratings

Statement *n* = 34	Strongly Disagree (%)	Disagree (%)	Neither Agree nor Disagree (%)	Agree (%)	Strongly Agree (%)
Time spent completing modules was well-matched with the knowledge gained.	0	0	1 (2.9)	15 (44.1)	18 (52.9)
The multi-media format improved the experience of completing the modules.	0	0	1 (2.9)	9 (26.5)	24 (70.6)
Content quality was consistent throughout the modules.	0	0	0	8 (23.5)	26 (76.5)
Modules were easy to follow.	0	0	0	8 (23.5)	26 (76.5)
Content was clear to facilitate learning.	0	0	0	9 (26.5)	25 (73.5)
Modules met my learning expectations	0	0	0	14 (41.2)	20 (58.8)
I would recommend the e-learning modules to colleagues.	0	0	1 (2.9)	8 (23.5)	25 (73.5)

While most respondents encountered no barriers to module completion, the most cited barrier (*n* = 4 or 11.4% of participants) was finding time to complete the modules. Participants answered open-ended questions about bias and provided feedback for future modifications. They felt that the modules were unbiased. However, two recognized that they lacked information on how to facilitate a session with an awareness of cultural differences and recommended this topic for future iterations. Three felt that including additional elements around cultural, systemic, and intergenerational trauma would create a more holistic and patient-focused program, which will be addressed in future program iterations.

### Highlights of learning experience

The topics in Table 4 were covered in the online modules and were seen as particularly valuable content by respondents.

**Table 4:** Topics in the modules that HCP participants highlighted as most important

Module Topic highlighted by participants	Definition
Traumatic re-enactments in adulthood (*n* = 10)	Ways trauma-based interpersonal patterns play out in current relationships or situations.
Emotional regulation strategies for patients (*n* = 10)	Models and skills on how to regulate emotional and physiological distress
Clinical pearls for HCPs facilitating future R&R groups (*n* = 6)	Tips for HCPs when working with content in a group format.
Dissociation – an explanation for HCPs on dissociative symptoms (*n* = 1)	Disconnecting from internal or external experiences as a protection against pain and distress.
Nourishment barriers as a form of self-protection (*n* = 2)	Difficulty taking in positive or healthy experiences or treating self with care and kindness.
Tension-reduction behaviours for patients (*n* = 1)	Various behaviours that are potentially harmful but are used to regulate trauma-related distress.

## Discussion

This study examined the feasibility, usability, and acceptability of the virtual, asynchronous CARE training program for healthcare providers. The CARE program consists of modules that educate HCPs about the psychoeducational content of the R&R group intervention for adults with a history of adverse childhood experiences, as well as how to deliver this content in a group setting. Despite high levels of prior e-learning experience, prior training in trauma-focused care, and over half having six or more years of experience in healthcare, participants reported that the modules continued to meet their learning needs, with all agreeing that the content met their expectations. These findings align with broader literature suggesting that even experienced HCPs value structured educational programs and may benefit from ongoing learning opportunities across their practice settings [55, 56]. Notably, most participants had relatively short tenures in their current positions (five years or less), suggesting that an easily accessible, asynchronous training program may be particularly well-suited to a healthcare environment characterized by high staff turnover. Training programs that utilize asynchronous or blended approaches can accommodate the dynamic nature of healthcare settings, provide ongoing training opportunities, and may support knowledge transfer even amidst personnel changes.

Feasibility of the CARE training program was supported by high levels of module completion and a time commitment that was generally manageable within routine clinical workflows. Nearly three-quarters of participants completed at least half of the modules, and almost two-thirds completed the full training, suggesting that the program could be delivered as intended in real-world healthcare settings. Most participants completed individual modules in under one hour, and the majority completed all eight modules within five to ten hours, indicating that the overall training burden was reasonable. Not unexpectedly, our study identified that the most reported barrier to attendance was a need for more time to complete the training modules, highlighting the importance of institutional support and paid time for employees to engage in training. This finding confirms the significant importance of protected time for online training programs seeking optimal implementation and sustainability [57, 58].

In terms of acceptability of the format and delivery of the CARE training program, participants reported positive experiences with the asynchronous, multimedia design. Integrating technology in education can effectively engage learners by incorporating multimedia content, such as video and audio recordings [59]. In the post-module evaluation, respondents appreciated the multimedia format of the asynchronous online modules, feeling that the time invested in module completion was well-balanced and appropriate to the knowledge gained. This positive reception of our asynchronous program may be influenced by the widespread adoption of virtual care during the COVID-19 pandemic, which likely enhanced participants’ familiarity and comfort with utilizing technology for learning. All participants reported prior engagement with online modules, indicating HCP’s growing acceptance of and integration of technology in healthcare education initiatives [13, 60]. These findings align with literature supporting the acceptability and usability of virtual platforms for delivering training on evidence-based treatments to HCPs with variable experience [61, 62].

With respect to acceptability of the training content, HCPs appreciated the psychoeducational nature of the CARE training program, emphasizing the valuable insights gained from experienced trauma therapists featured in the modules and the integration of emotion regulation skills and tension-reduction behaviours within the program. These results align with existing literature supporting the acceptability and usefulness of psychoeducational interventions in mental health, including those used by HCPs in diverse settings [63, 64]. Stepped care refers to matching intervention intensity to patient needs; the CARE training prepares HCPs to deliver a lower-intensity psychoeducational intervention that aligns with the early steps of stepped care.

Training that targets diverse HCPs, rather than a single profession, can provide an opportunity to bridge gaps and communication divides between different professional perspectives by providing shared knowledge and a common language learned [65]. In this study, usability of the CARE training program was supported by participants’ reports that the modules were easy to follow and navigate, content was clear to facilitate learning, and no significant technical or accessibility barriers were encountered. Participants described the program as practical to use and perceived it as unbiased. Suggestions for additional content on facilitating sessions that consider cultural differences, systemic issues, and intergenerational trauma will be addressed in the program’s next iteration, followed by consultation with key stakeholders. Our findings are supported by research demonstrating the effectiveness of web-based training programs in engaging multidisciplinary audiences, even without tailoring the content for specific provider roles [66, 67].

A strength of our study is the inclusion of HCPs from six different healthcare organizations, broadening our findings’ generalizability. However, the community organizations involved in the study were not randomly selected; they approached us for training, which may have introduced selection bias. In addition, provider-type data was not collected in this feasibility phase, limiting our ability to examine differences across disciplines or roles. Provider-type data will be collected in the next phase, enabling examination of role-specific engagement patterns. We also did not collect data from non-completers, which introduces the possibility of completion bias. Additionally, completion rates for post-module questions (*n* = 40) were lower than pre-module questions (*n* = 62), potentially impacting the reliability and validity of our data. Systematic reviews have noted that more research is required to gain a better understanding of how to optimize the design, development, and implementation of online interventions [68–70]. We did not explore the attitudes and acceptability of the program in organizational leaders/management beyond their sanctioning of employees’ participation in the study; this will be important to factor into a future implementation phase of this study. However, we anticipate offering organizations access to the training at no cost in the next phase of this study, which may add to its appeal to future partners.

## Conclusion

Our study strengthens the existing literature’s emphasis on the need for accessible, affordable, and scalable trauma-focused training on psychoeducational interventions [7, 71]. HCPs across six healthcare organizations responded positively to our virtual CARE training program’s asynchronous, multimedia, psychoeducational content. Our results suggest that the CARE training program offers a feasible and engaging approach to trauma-focused psychoeducational training. Future phases of this study will include post-training confidence and knowledge outcomes and will examine effectiveness and implementation.

## Supplementary Information

Supplementary Material 1.

## Data Availability

The datasets used and/or analysed during the current study are available from the corresponding author on reasonable request.

## Abbreviations

HCP: Healthcare Provider
TTP: Trauma Therapy Program
WCH: Women’s College Hospital
R&R: Resourced & Resilient

## References

[1] Joshi D, Raina P, Tonmyr L, MacMillan HL, Gonzalez A. Prevalence of adverse childhood experiences among individuals aged 45 to 85 years: a cross-sectional analysis of the Canadian longitudinal study on aging. CMAJ Open. 2021;9(1):E158–66. 10.9778/cmajo.20200064 PMC8034300 33653771

[2] Sucich J, Breitbart V, Williams S, Sanichar N, Candelaria-Arce E, Frankle WG, et al. Prevalence of childhood trauma in a community-based mental health clinic. Community Ment Health J. 2023;59(6):1136–49. 10.1007/s10597-023-01094-1 36752932

[3] Wainberg ML, Scorza P, Shultz JM, Helpman L, Mootz JJ, Johnson KA, et al. Challenges and opportunities in global mental health: a research-to-practice perspective. Curr Psychiatry Rep. 2017;19(5):28. 28425023 10.1007/s11920-017-0780-z PMC5553319

[4] Alcalá HE, Valdez-Dadia A, von Ehrenstein OS. Adverse childhood experiences and access and utilization of health care. J Public Health (Oxf). 2018;40(4):684–92. 29182751 10.1093/pubmed/fdx155 PMC6306082

[5] Kazlauskas E. Challenges for providing health care in traumatized populations: barriers for PTSD treatments and the need for new developments. Glob Health Action. 2017;10(1):1322399. 28562198 10.1080/16549716.2017.1322399 PMC5496089

[6] Finch J, Ford C, Grainger L, Meiser-Stedman R. A systematic review of the clinician related barriers and facilitators to the use of evidence-informed interventions for post traumatic stress. J Affect Disord. 2020;263:175–86. 31818775 10.1016/j.jad.2019.11.143

[7] Kumar SA, Brand BL, Courtois CA. The need for trauma training: clinicians’ reactions to training on complex trauma. Psychol Trauma. 2022;14(8):1387–94. 31580137 10.1037/tra0000515

[8] Ghafoori B, Fisher D, Korosteleva O, Hong MA, Randomized. Controlled pilot study of a single-session psychoeducation treatment for Urban, culturally diverse, trauma-exposed adults. J Nerv Ment Dis. 2016;204(6):421–30. 27027660 10.1097/NMD.0000000000000512 PMC4884137

[9] Brouzos A, Vatkali E, Mavridis D, et al. Psychoeducation for adults with Post-Traumatic stress symptomatology: a systematic review and meta-analysis. J Contemp Psychother. 2022;52:155–64.

[10] Hartzler B, Hinde J, Lang S, Correia N, Yermash J, Yap K, et al. Virtual training is more cost-effective than in-person training for preparing staff to implement contingency management. J Technol Behav Sci. 2023:8:255–64. 10.1007/s41347-022-00283-1 PMC9553630 36246531

[11] Bajra R, Frazier W, Graves L, Jacobson K, Rodriguez A, Theobald M, et al. Feasibility and acceptability of a US National telemedicine curriculum for medical students and residents: multi-institutional cross-sectional study. JMIR Med Educ. 2023;9:e43190. 37155241 10.2196/43190 PMC10203924

[12] Vallée A, Blacher J, Cariou A, Sorbets E. Blended learning compared to traditional learning in medical education: systematic review and meta-analysis. J Med Internet Res. 2020;22(8):e16504. 32773378 10.2196/16504 PMC7445617

[13] Du L, Zhao L, Xu T, Wang Y, Zu W, Huang X, et al. Blended learning vs traditional teaching: the potential of a novel teaching strategy in nursing education - a systematic review and meta-analysis. Nurse Educ Pract. 2022;63:103354. 35580368 10.1016/j.nepr.2022.103354

[14] Hwang NK, Shim SH, Cheon HW. Digital learning designs in occupational therapy education: a scoping review. BMC Med Educ. 2023;23(1):7. 36604723 10.1186/s12909-022-03955-x PMC9817377

[15] Chawla N, Gyawali S, Sharma P, Balhara YPS. Internet-based learning for professionals in addiction psychiatry: a scoping review. Indian J Psychol Med. 2022;44(4):325–31. 35949641 10.1177/02537176221082897 PMC9301747

[16] Clair V, Atkinson K, Musau A, Mutiso V, Bosire E, Gitonga I, et al. Implementing and sustaining brief addiction medicine interventions with the support of a quality improvement Blended-eLearning course: learner experiences and meaningful outcomes in Kenya. Int J Ment Health Addict. 2022;20(6):3479–500. 35634518 10.1007/s11469-022-00781-6 PMC9126625

[17] Sinclair PM, Kable A, Levett-Jones T, Booth D. The effectiveness of Internet-based e-learning on clinician behaviour and patient outcomes: a systematic review. Int J Nurs Stud. 2016;57:70–81. 27045566 10.1016/j.ijnurstu.2016.01.011

[18] Talley R, Chiang IC, Covell NH, Dixon L. Comparative initial and sustained engagement in Web-based training by behavioral healthcare providers in New York state. J Technol Behav Sci. 2018;3(2):41–8. 29732398 10.1007/s41347-017-0027-1 PMC5931788

[19] Naslund JA, Shidhaye R, Patel V. Digital technology for Building capacity of nonspecialist health workers for task sharing and scaling up mental health care globally. Harv Rev Psychiatry. 2019;27(3):181–92. 30958400 10.1097/HRP.0000000000000217 PMC6517068

[20] Davis LL, Schein J, Cloutier M, Gagnon-Sanschagrin P, Maitland J, Urganus A, et al. The economic burden of posttraumatic stress disorder in the United States from a societal perspective. J Clin Psychiatry. 2022;83(3):21m14116. 10.4088/JCP.21m14116 35485933

[21] Schnyder U, Bryant RA, Ehlers A, Foa EB, Hasan A, Mwiti G, et al. Culture-sensitive psychotraumatology. Eur J Psychotraumatol. 2016;7:31179. 27473520 10.3402/ejpt.v7.31179 PMC5055610

[22] Keynejad R, Spagnolo J, Thornicroft G. WHO mental health gap action programme (mhGAP) intervention guide: updated systematic review on evidence and impact. Evid Based Ment Health. 2021;24(3):124–30. 33903119 10.1136/ebmental-2021-300254 PMC8311089

[23] Spaulding EM, Marvel FA, Jacob E, Rahman A, Hansen BR, Hanyok LA, et al. Interprofessional education and collaboration among healthcare students and professionals: a systematic review and call for action. J Interprof Care. 2021;35(4):612–21. 31865823 10.1080/13561820.2019.1697214 PMC7305974

[24] Patel V, Naslund JA, Wood S, Patel A, Chauvin JJ, Agrawal R, et al. EMPOWER: toward the global dissemination of psychosocial interventions. Focus (Am Psychiatr Publ). 2022;20(3):301–6. 37021040 10.1176/appi.focus.20220042 PMC10071408

[25] Rahman A, Hamdani SU, Awan NR, Bryant RA, Dawson KS, Khan MF, et al. Effect of a multicomponent behavioral intervention in adults impaired by psychological distress in a conflict-affected area of pakistan: a randomized clinical trial. JAMA. 2016;316(24):2609–17. 27837602 10.1001/jama.2016.17165

[26] Coventry PA, Meader N, Melton H, Temple M, Dale H, Wright K, et al. Psychological and pharmacological interventions for posttraumatic stress disorder and comorbid mental health problems following complex traumatic events: systematic review and component network meta-analysis. PLoS Med. 2020;17(8):e1003262. 32813696 10.1371/journal.pmed.1003262 PMC7446790

[27] Maercker A, Cloitre M, Bachem R, Schlumpf YR, Khoury B, Hitchcock C, et al. Complex post-traumatic stress disorder. Lancet. 2022;400(10345):60–72. 35780794 10.1016/S0140-6736(22)00821-2

[28] Dolan N, Simmonds-Buckley M, Kellett S, Siddell E, Delgadillo J. Effectiveness of stress control large group psychoeducation for anxiety and depression: systematic review and meta-analysis. Br J Clin Psychol. 2021;60(3):375–99. 33822376 10.1111/bjc.12288

[29] Bighelli I, Rodolico A, García-Mieres H, Pitschel-Walz G, Hansen WP, Schneider-Thoma J, et al. Psychosocial and psychological interventions for relapse prevention in schizophrenia: a systematic review and network meta-analysis. Lancet Psychiatry. 2021;8(11):969–80. 34653393 10.1016/S2215-0366(21)00243-1

[30] Miklowitz DJ, Efthimiou O, Furukawa TA, Scott J, McLaren R, Geddes JR, et al. Adjunctive psychotherapy for bipolar disorder: a systematic review and component network meta-analysis. JAMA Psychiatry. 2021;78(2):141–50. 33052390 10.1001/jamapsychiatry.2020.2993 PMC7557716

[31] Ekhtiari H, Rezapour T, Aupperle RL, Paulus MP. Neuroscience-informed psychoeducation for addiction medicine: a neurocognitive perspective. Prog Brain Res. 2017;235:239–64. 29054291 10.1016/bs.pbr.2017.08.013 PMC5771228

[32] Magill M, Martino S, Wampold B. The principles and practices of psychoeducation with alcohol or other drug use disorders: a review and brief guide. J Subst Abuse Treat. 2021;126:108442. 34116812 10.1016/j.jsat.2021.108442 PMC8197778

[33] Buizza C, Candini V, Ferrari C, Ghilardi A, Saviotti FM, Turrina C, et al. The long-term effectiveness of psychoeducation for bipolar disorders in mental health Services. A 4-year follow-Up study. Front Psychiatry. 2019;10:873. 31849726 10.3389/fpsyt.2019.00873 PMC6901938

[34] Rabelo JL, Cruz BF, Ferreira JDR, Viana BM, Barbosa IG. Psychoeducation in bipolar disorder: a systematic review. World J Psychiatry. 2021;11(12):1407–24. 35070785 10.5498/wjp.v11.i12.1407 PMC8717031

[35] Burns P, Kellett S, Donohoe G. Stress control as a large group psychoeducational intervention at step 2 of IAPT services: acceptability of the approach and moderators of effectiveness. Behav Cogn Psychother. 2016;44(4):431–43. 26365006 10.1017/S1352465815000491

[36] Banbury A, Nancarrow S, Dart J, Gray L, Parkinson L. Telehealth interventions delivering Home-based support group videoconferencing: systematic review. J Med Internet Res. 2018;20(2):e25. 29396387 10.2196/jmir.8090 PMC5816261

[37] Sweet CMC, Li EJ, Sagui-Henson S, Chamberlain CEW, Altman M. Impact of online group psychoeducation and support sessions on receptivity towards digital mental health care during the COVID-19 pandemic: a pilot study. J Technol Behav Sci. 2022;8:216–24. 10.1007/s41347-022-00281-3 PMC9510187 36189429

[38] Ross DC, McCallum N, Truuvert AK, Butt A, Behdinan T, Rojas D, et al. The development and evaluation of a virtual, asynchronous, trauma-focused treatment program for adult survivors of childhood interpersonal trauma. J Ment Health. 2024:33(5):566–75. 10.1080/09638237.2024.2332797 38572918

[39] Behdinan T, Truuvert AK, Adekunte A, McCallum N, Vigod SN, Butt A , et al. The Trauma PORTAL-A blended e-Health intervention for survivors of childhood interpersonal trauma: an open-label pilot study. Telemed Rep. 2024;5(1):195–204. 10.1089/tmr.2024.0020 PMC11286000 39081455

[40] Ross DC. The Trauma Portal Project: a Virtual, Asynchronous Treatment for Interpersonal Trauma 2022 [CT05670405]. Available from: https://clinicaltrials.gov/study/NCT05670405.

[41] Brown VB, Najavits LM, Cadiz S, Finkelstein N, Heckman JP, Rechberger E. Implementing an evidence-based practice: seeking safety group. J Psychoact Drugs. 2007;39(3):231–40. 10.1080/02791072.2007.10400609 18159776

[42] Watkins LE, Sprang KR, Rothbaum BO, Treating PTSD. A review of evidence-based psychotherapy interventions. Front Behav Neurosci. 2018;12:258. 30450043 10.3389/fnbeh.2018.00258 PMC6224348

[43] Karatzias T, Cloitre M. Treating adults with complex posttraumatic stress disorder using a modular approach to treatment: rationale, evidence, and directions for future research. J Trauma Stress. 2019;32(6):870–6. 31730720 10.1002/jts.22457

[44] Carpenter JK, Andrews LA, Witcraft SM, Powers MB, Smits JAJ, Hofmann SG. Cognitive behavioral therapy for anxiety and related disorders: a meta-analysis of randomized placebo-controlled trials. Depress Anxiety. 2018;35(6):502–14. 29451967 10.1002/da.22728 PMC5992015

[45] Charney ME, Chow L, Jakubovic RJ, Federico LE, Goetter EM, Baier AL, et al. Training community providers in evidence-based treatment for PTSD: outcomes of a novel consultation program. Psychol Trauma. 2019;11(7):793–801. 30688511 10.1037/tra0000427

[46] Bohus M, Kleindienst N, Hahn C, Müller-Engelmann M, Ludäscher P, Steil R, et al. Dialectical behavior therapy for posttraumatic stress disorder (DBT-PTSD) compared with cognitive processing therapy (CPT) in complex presentations of PTSD in women survivors of childhood abuse: a randomized clinical trial. JAMA Psychiatry. 2020;77(12):1235–45. 32697288 10.1001/jamapsychiatry.2020.2148 PMC7376475

[47] Goldsmith RE, Gerhart JI, Chesney SA, Burns JW, Kleinman B, Hood MM. Mindfulness-based stress reduction for posttraumatic stress symptoms: building acceptance and decreasing shame. J Evid Based Complement Altern Med. 2014;19(4):227–34. 10.1177/2156587214533703 24812075

[48] Classen CC, Hughes L, Clark C, Hill Mohammed B, Woods P, Beckett B. A pilot RCT of a body-oriented group therapy for complex trauma survivors: an adaptation of sensorimotor psychotherapy. J Trauma Dissociation. 2021;22(1):52–68. 32419670 10.1080/15299732.2020.1760173

[49] Pong R, Tham YC, Lum E. Current implementation of digital health in chronic disease management: scoping review. J Med Internet Res. 2024;26:e53576. 39666972 10.2196/53576 PMC11671791

[50] Klaic M, Kapp S, Hudson P, Chapman W, Denehy L, Story D, et al. Implementability of healthcare interventions: an overview of reviews and development of a conceptual framework. Implement Sci. 2022;17(1):10. 35086538 10.1186/s13012-021-01171-7 PMC8793098

[51] Park SY, Nicksic Sigmon C, Boeldt D. A framework for the implementation of digital mental health interventions: the importance of feasibility and acceptability research. Cureus. 2022;14(9):e29329. 36277565 10.7759/cureus.29329 PMC9580609

[52] Subba P, Shrestha P, Rahman A, Luitel N, Waqas A, Sikander S. Feasibility and acceptability of community-based psychosocial interventions delivered by nonspecialists for perinatal common mental disorders: a systematic review using an implementation science framework. Glob Ment Health (Camb). 2025;12:e54. 40556715 10.1017/gmh.2025.10010 PMC12186571

[53] Arora AK, Rodriguez C, Carver T, Teper MH, Rojas-Rozo L, Schuster T. Evaluating usability in blended learning programs within health professions education: a scoping review. Med Sci Educ. 2021;31(3):1213–46. 34031640 10.1007/s40670-021-01295-x PMC8133057

[54] Proctor EK, Bunger AC, Lengnick-Hall R, Gerke DR, Martin JK, Phillips RJ, et al. Ten years of implementation outcomes research: a scoping review. Implement Sci. 2023;18(1):31. 37491242 10.1186/s13012-023-01286-z PMC10367273

[55] Appleton R, Barnett P, Vera San Juan N, Tuudah E, Lyons N, Parker J, et al. Implementation strategies for telemental health: a systematic review. BMC Health Serv Res. 2023;23(1):78. 36694164 10.1186/s12913-022-08993-1 PMC9873395

[56] Kimura R, Matsunaga M, Barroga E, Hayashi N. Asynchronous e-learning with technology-enabled and enhanced training for continuing education of nurses: a scoping review. BMC Med Educ. 2023;23(1):505. 37442970 10.1186/s12909-023-04477-w PMC10339492

[57] Schueller SM, Torous J. Scaling evidence-based treatments through digital mental health. Am Psychol. 2020;75(8):1093–104. 33252947 10.1037/amp0000654 PMC7709142

[58] Ross B, Penkunas MJ, Maher D, Certain E, Launois P. Evaluating results of the implementation research MOOC using kirkpatrick’s four-level model: a cross-sectional mixed-methods study. BMJ Open. 2022;12(5):e054719. 10.1136/bmjopen-2021-054719 PMC9066485 35504633

[59] Choe RC, Scuric Z, Eshkol E, Cruser S, Arndt A, Cox R, et al. Student satisfaction and learning outcomes in asynchronous online lecture videos. CBE Life Sci Educ. 2019;18(4):ar55. 31675279 10.1187/cbe.18-08-0171 PMC6829069

[60] Chung-Lee L, Catallo C. A new approach to digital health? Virtual COVID-19 care: a scoping review. Digit Health. 2023;9:20552076231152171. 36798886 10.1177/20552076231152171 PMC9926398

[61] Vona P, Wilmoth P, Jaycox LH, McMillen JS, Kataoka SH, Wong M, et al. A web-based platform to support an evidence-based mental health intervention: lessons from the CBITS web site. Psychiatr Serv. 2014;65(11):1381–4. 25124275 10.1176/appi.ps.201300512 PMC5361218

[62] Soll D, Fuchs R, Mehl S. Teaching cognitive behavior therapy to postgraduate health care professionals in times of COVID 19 - An asynchronous blended learning environment proved to be non-inferior to In-Person training. Front Psychol. 2021;12:657234. 34646190 10.3389/fpsyg.2021.657234 PMC8504537

[63] Eichfeld C, Farrell D, Mattheß M, Bumke P, Sodemann U, Ean N, et al. Trauma stabilisation as a sole treatment intervention for Post-Traumatic stress disorder in Southeast Asia. Psychiatr Q. 2019;90(1):63–88. 30267358 10.1007/s11126-018-9598-z PMC6426800

[64] Sarkhel S, Singh OP, Arora M. Clinical practice guidelines for psychoeducation in psychiatric disorders general principles of psychoeducation. Indian J Psychiatry. 2020;62(Suppl 2):S319–23. 32055073 10.4103/psychiatry.IndianJPsychiatry_780_19 PMC7001357

[65] Schot E, Tummers L, Noordegraaf M. Working on working together. A systematic review on how healthcare professionals contribute to interprofessional collaboration. J Interprof Care. 2020;34(3):332–42. 31329469 10.1080/13561820.2019.1636007

[66] Kodweis KR, Hall EA, Renfro CP, Thomas-Gosain N, Lennon-Dearing R, Walker JK, et al. Successful development and implementation of a large virtual interprofessional education activity applying the social determinants of health. Pharm (Basel). 2022;10(6):157. 10.3390/pharmacy10060157 PMC9780877 36548313

[67] Nguyen D, Tupas KD, Thammasitboon S. Evolution of a continuing professional development program based on a community of practice model for health care professionals in resource-limited settings. J Contin Educ Health Prof. 2023;44(1):58–63. 10.1097/CEH.0000000000000505 37141179

[68] Côté-Boileau É, Denis JL, Callery B, Sabean M. The unpredictable journeys of spreading, sustaining and scaling healthcare innovations: a scoping review. Health Res Policy Syst. 2019;17(1):84. 31519185 10.1186/s12961-019-0482-6 PMC6744644

[69] Gan DZQ, McGillivray L, Larsen ME, Christensen H, Torok M. Technology-supported strategies for promoting user engagement with digital mental health interventions: a systematic review. Digit Health. 2022;8:20552076221098268. 35677785 10.1177/20552076221098268 PMC9168921

[70] Philippe TJ, Sikder N, Jackson A, Koblanski ME, Liow E, Pilarinos A, et al. Digital health interventions for delivery of mental health care: systematic and comprehensive meta-review. JMIR Ment Health. 2022;9(5):e35159. 35551058 10.2196/35159 PMC9109782

[71] Connolly SM, Vanchu-Orosco M, Warner J, Seidi PA, Edwards J, Boath E, et al. Mental health interventions by Lay counsellors: a systematic review and meta-analysis. Bull World Health Organ. 2021;99(8):572–82. 34354312 10.2471/BLT.20.269050 PMC8319860

