# Supplementary material for: Feasibility, usability, and acceptability of the asynchronous trauma-focused CARE training program for healthcare providers: a cross-sectional study
Source: BMC Med Educ. 2026 Feb 18;26:485. doi: 10.1186/s12909-026-08794-8 (PMC13020217; doi:10.1186/s12909-026-08794-8)
Supplement: Supplementary file 1 — Supplementary Material 1. [file 12909_2026_8794_MOESM1_ESM.docx]

**Supplementary File #1**

**Pre-Test Evaluation Survey**

Background:

1. How long have you been working in your current position?
2. Less than 1 year
3. 1-5 years
4. 6-10 years
5. 11-15 years
6. 16+ years
7. How long have you been working in the healthcare field?
8. Less than 1 year
9. 1-5 years
10. 6-10 years
11. 10-20 years
12. 20+ years
13. Have you completed e-learning modules in the past?
14. Yes
15. No
16. Have you done any previous training on delivering trauma-focused care?
17. Yes
18. No

**Post-Test Evaluation Survey**

| **Likert-scale Acceptability & Usability Questions** | **Strongly Agree** | **Agree** | **Neither Agree nor Disagree** | **Disagree** | **Strongly Disagree** |
| --- | --- | --- | --- | --- | --- |
| 1. The quality of the content was consistent throughout the e-learning modules. |  |  |  |  |  |
| 2. The e-learning modules on the website were easy to navigate. |  |  |  |  |  |
| 3. I would recommend the e-learning modules to colleagues. |  |  |  |  |  |
| 4. The time required to complete the e-learning modules was consequent with the knowledge gained |  |  |  |  |  |
| The overall content was clear in facilitating my learning |  |  |  |  |  |
| The overall content of the e-learning modules met my expectations |  |  |  |  |  |
| The multimedia format of the modules improved my overall experience of completing the e-learning modules. |  |  |  |  |  |

| **Open-Ended Acceptability & Usability** | **Open-ended answer** |
| --- | --- |
| How could we improve the overall learning experience? |  |
| How could specific e-learning modules be improved? |  |
| Did you experience any technical difficulties on our website? If so, please explain. |  |
| Were the training modules free from bias? If not, please elaborate. |  |
| Were there any concerns that you had about accessibility (e.g., audio-visual aids, colour schemes, navigation)? If so, please provide details. |  |
| Can you tell us 3 things that were the biggest highlights for you on this e-learning? |  |
| Do you have any other comments, feedback or suggestions? |  |

**Feasibility**

| **Open-ended Feasibility Questions** | **Open-ended answer** |
| --- | --- |
| On average, how much time did it take for you to complete all 8 modules |  |
| Were there any barriers to completing the e-learning modules? |  |

| **Likert-style categorical Feasibility Question** |  |  |  |  |
| --- | --- | --- | --- | --- |
| On average, how much time did it take for you to complete each module? | Less than 30 minutes | 30 minutes | 1-2 hours | 2+ hours |

**How would you rate your confidence level on co-facilitating a trauma-focused group with your clients:**

|  | Very Confident | Confident | Somewhat Confident | Not Confident |
| --- | --- | --- | --- | --- |
| Prior to completing the e-learning modules |  |  |  |  |
| After completing the e-learning modules |  |  |  |  |
